# Research Progress on Structural Modification of Effective Antitumor Active Ingredients in Licorice

**DOI:** 10.3390/molecules28155855

**Published:** 2023-08-03

**Authors:** Chi Liu, Qing Ma, Guangfei Gong, Fengyan Su

**Affiliations:** College of Chinese Medicinal Material, Jilin Agricultural University, 2888 Xincheng Street, Changchun 130118, China; liuchi0222@163.com (C.L.); mq15141834269@163.com (Q.M.); 16696417075@163.com (G.G.)

**Keywords:** licorice, antitumor, chemical composition, chemical modifications

## Abstract

Licorice, a widely used traditional Chinese medicine, contains more than 300 flavonoids and more than 20 triterpenoids, which have potential medicinal value and can prevent the growth of tumor cells by blocking the cell cycle, affecting the regulation of the apoptosis gene of tumor cells, and inhibiting tumor cell angiogenesis. However, many of the compounds in licorice still have the drawbacks of poor solubility, significant toxic side effects, and low antitumor activity. This article reviews the structural modification of effective antitumor active ingredients in licorice, thus providing a theoretical basis for further investigation of licorice and the development of new antitumor drugs.

## 1. Introduction

A serious condition that endangers human health is cancer. Licorice is the dried root and rhizome of the legumes *Glycyrrhiza uralensis Fisch*., *Glycyrrhiza inflata Bat*., and *Glycyrrhiza glabra* L. [1], which has the effect of tonifying the spleen and invigorating qi, clearing heat and detoxification, acting as an expectorant and cough suppressant, reducing pain, and harmonizing various medicines [2,3]. Licorice has both direct and indirect effects on tumors, including disruption of the cell cycle [4], induction of apoptosis and autophagy [5,6], inhibition of cancer cell migration and spread [7], regulation of immunity [8], etc. It also benefits from a long-lasting action time, a wide range of targets, multiple pathways, and minimal toxic side effects. But, many licorice monomeric compounds still suffer from poor solubility, significant toxic side effects, and weak antitumor effectiveness. However, plenty of licorice’s monomeric compounds still suffer from issues such poor solubility, significant toxic side effects, and weak antitumor effectiveness when used in clinical settings. In light of these shortcomings, numerous research teams, both domestically and internationally, use cutting-edge science and technology to chemically alter the antitumor components of licorice in order to produce derivatives with high biological activity and good solubility and create novel antitumor medications. To provide a theoretical foundation for the continued development and application of licorice, this paper primarily analyzes the scientific progress of structural alterations of helpful antitumor chemical components in licorice.

## 2. Ingredient Research

### 2.1. Flavonoids

**Figure 1:**
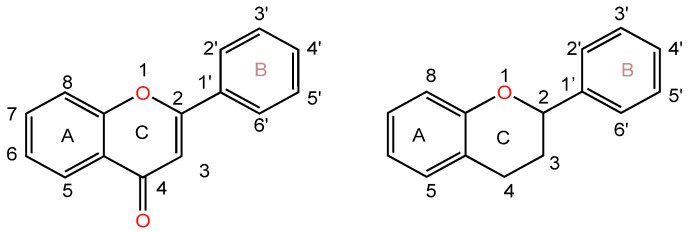
2-Phenylchromone structure and C6-C3-C6 basic skeleton.

The basic parent nucleus of licorice flavonoids is 2-phenylchromonone, which refers to a series of compounds formed by two benzene rings (A and B rings) connected to each other by three carbon atoms. Most have the basic skeleton of C6-C3-C6 (Figure 1).

#### 2.1.1. Flavonoids

**Figure 2:**
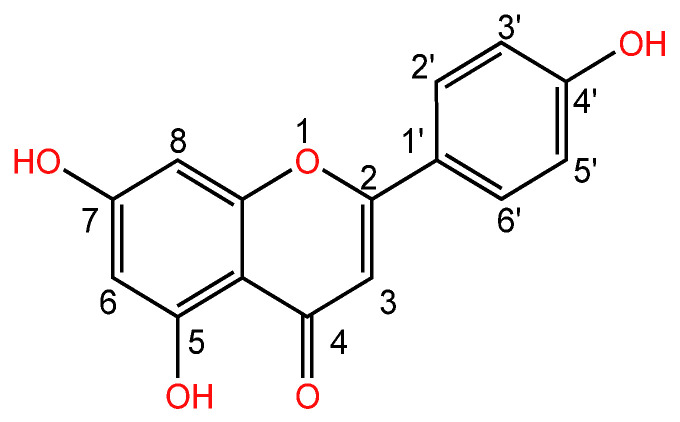
The structure of apigenin.

**Scheme 1:**
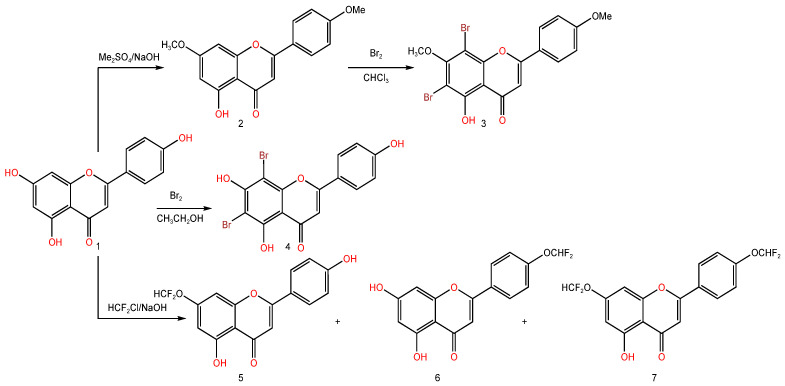
Synthetic route of apigenin etherified derivatives.

**Scheme 2:**
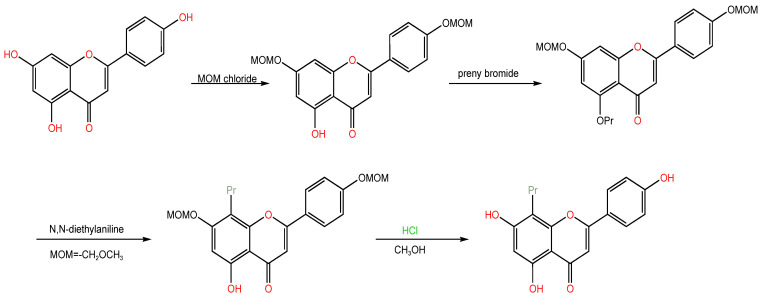
Synthetic route of 8-isoprenylapigenin.

**Scheme 3:**
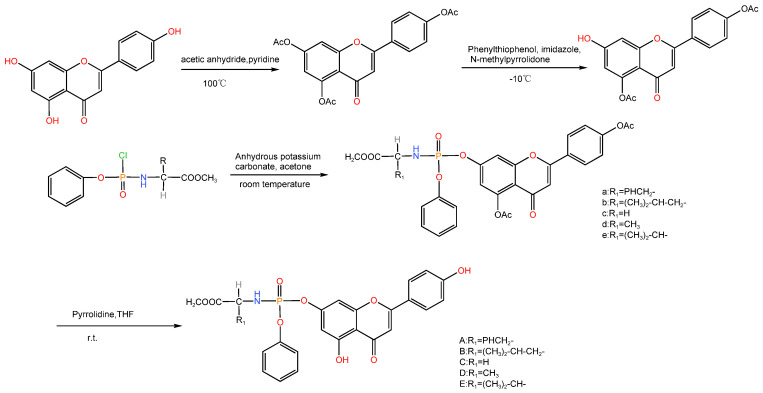
Route of synthesis of apigenin esterified derivatives.

**Scheme 4:**
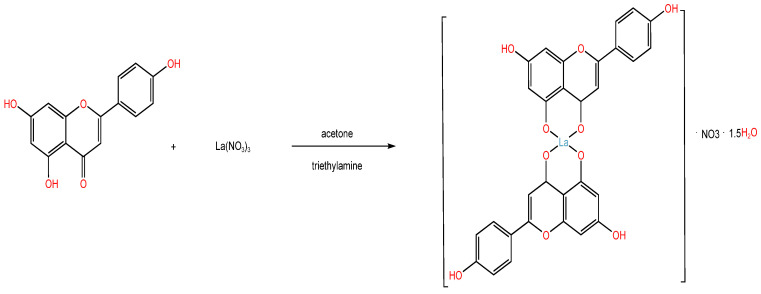
Apigenin complex synthesis route.

The following 15 different kinds of flavonoids have the most frequently found in licorice (Table 1), while apigenin (Figure 2) has received more research attention for its structural changes and antitumor abilities. Following a review of the pertinent literature, it emerged that apigenin’s structural change focused mainly on the following categories:

(1) Etherification: Xiang et al. [17] used apigenin as raw materials to obtain six apigenin derivatives by introducing methyl ether and difluoromethyl ether at the 7th position of the A ring, difluoromethyl ether at the 4′ position of the B ring, difluoromethyl ether in the 7′ position of the A ring and 4′ position of the B ring at the same time, and bromine atoms at the 6th and 8th positions of the A ring at the same time, and the anti-liver cancer activity of the derivatives was studied in vitro (Scheme 1). The results showed that compound **3** (6,8-dibromo-7,4-dimethoxy-5-hydroxyflavone) had the strongest anti-liver cancer activity, with a potency 12.09 times that of the lead apigenin and 11.05 times that of the chemotherapy drug 5-FU, and the structure–activity relationship analysis speculated that it may be related to its simultaneous methyl etherification and bromination, and Cardenas et al [18] also found that the introduction of Cl or Br atoms at the C6 position of the flavonoid A ring can enhance its antitumor activity.

(2) Alkylation: According to Nikaido, T. et al. [19], the presence of isoprene groups in flavonoids greatly increased their lipophilicity, which can enable compound molecules to exhibit strong affinity for biofilms, which in turn can cause a significant increase in biological activity. The structure of apigenin includes three hydroxyl groups, namely, 4′, 5, and 7, and the activities of the various locations vary. Daskiewicz, J.B. et al. [20] protected phenolic hydroxyl groups at position 7 and 4′ with chloromethyl methyl ether, phenolic hydroxyl etherification and the introduction of isoprene at position C-5, and then they rearranged σ migration, migrated the 5-position isoprene group to position 8 on the skeleton, and finally deprotected to obtain 8-isoprenylapigenin (Scheme 2). Activity tests showed that the introduction of isoprene at the 8th position of apigenin could significantly inhibit the proliferation of HT-29 in human colon cancer cells and promote their apoptosis.

(3) Esterification: Aminophosphate is a phosphorus-containing analogue of natural amino acids; the introduction of amino phosphate can often enhance the antitumor activity of the parent drug, and some phosphoramide nitrogen mustard derivatives, such as cyclophosphamide, have been applied because of their good anticancer activity [21]. In order not to affect the amide bonds and ester bonds in the amino phosphate structure introduced by apigenin, Han, T [22] chose acetylation under the action of pyridine and acetic anhydride to protect the 4′, 5, and 7 hydroxyl groups, because the hydroxyl group on the 7th position is in the 4-position carbonyl group, the electron-absorbing effect of the carbonyl group enhanced the 7-hydroxyl acidity, so compared with other hydroxyl groups, it was easier to remove the 7-position hydroxyl group, obtain 4′, obtain 5-diacetylapigenin at its 7th position deprotector, and remove 4′. The results show that the five compounds could inhibit tumor cell proliferation to varying degrees, showing good tumor proliferation inhibition activity, especially B (substituent is leucine), D (substituent is alanine), and E (substituent is valine), with better activity, and the IC50 values were 25, 26.2, and 21 μmol/L, respectively, which has certain theoretical and practical significance for the development of drugs for the treatment of cervical cancer (Scheme 3).

(4) Formation of complexes: Modern research has demonstrated that rare earth metals have unique electronic structures, bonding properties, and high coordination numbers. By forming complexes with naturally occurring substances that have pharmacological activity, these metals can enhance or expand the pharmacological effects of the original ligand, and their toxicity is significantly decreased. Lanthanum nitrate and apigenin were used to create the lanthanum apigenin complex for the first time. The complex’s antitumor activity was tested on the cervical cancer cell line Hela, and the results revealed that it significantly inhibited cell proliferation when the concentration was higher than 30 ug/mL. The inhibitory impact grows over the same period of time as the compound concentration increases; at the same concentration point, the inhibitory effect rose as the complex’s action duration grew longer. It has greater biological activity than the ligand apigenin [23] (Scheme 4).

#### 2.1.2. Flavonols

**Figure 3:**
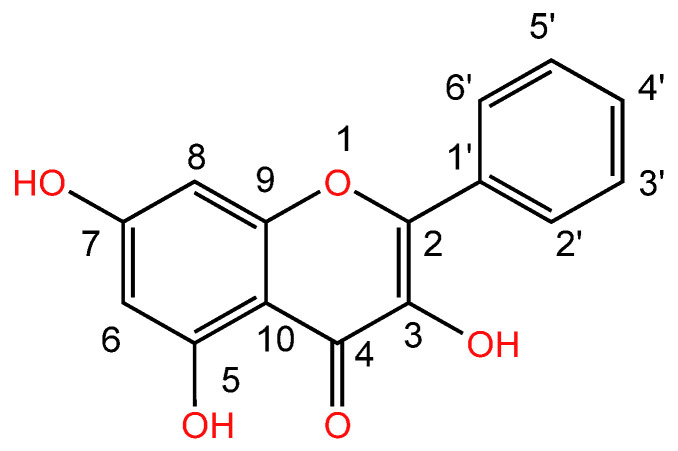
The structure of galangin.

**Figure 4:**
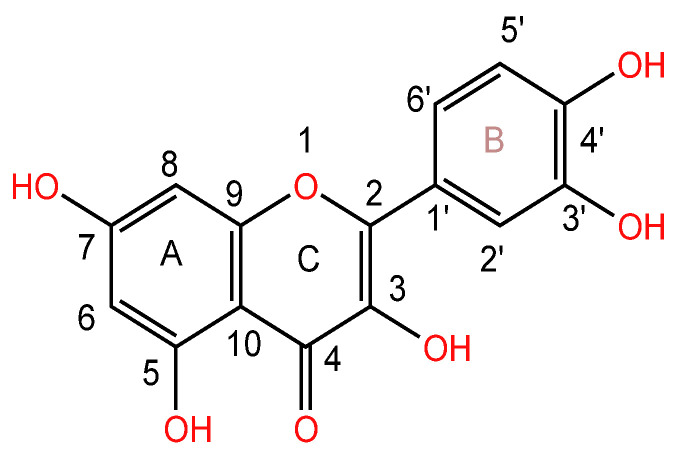
The structure of quercetin.

Currently, licorice contains mostly the following eight flavonols (Table 2), and in the study of structural modification of its antitumor components, galangin (Figure 3) and quercetin (Figure 4) have been reported more.

**Scheme 5:**
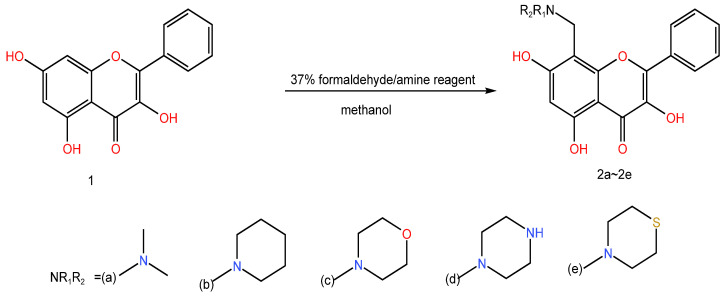
Synthetic route of galangin nitrogenous derivatives.

**Figure 5:**
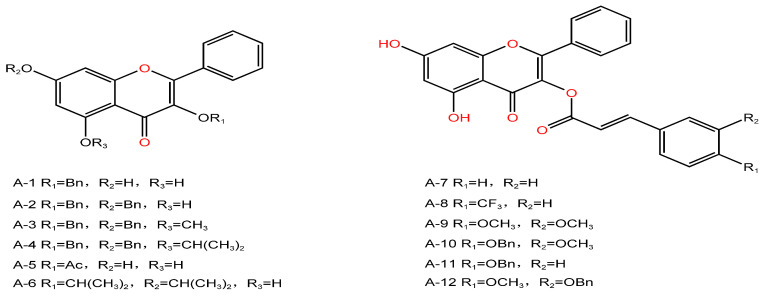
Galangin 3, 5, and 7 substituent derivatives.

Fields of exploration into galangin’s structural change are outlined in the following order:

(1) At position 8, nitrogenous basic groups are inserted: With galangin as the starting material, Luo, Y et al. [29] introduced several nitrogen-containing heterocyclic compounds at the 8th position of its structure through the Mannich reaction (Scheme 5). The product’s antitumor activity was then evaluated using the MTT method, and the results shown that compound **2c** and **2d** had substantially higher proliferative inhibitory activity than galangin (IC50: 11.37 and 13.57 μmol/L) on human prostate cancer PC-3M. It was concluded that the addition of galangin 8-position nitrogenous heterocycles may be responsible for the augmentation of derivative activity after compound **2d**’s proliferation inhibitory activity on human colon cancer LOVO cell lines had been raised 5.22-fold.

(2) Introduction of substituents at positions 3, 5, 7: Zhao, L et al. [30] conducted research on the effect of hindering the proliferation of human myeloid leukemia K562 cells through the inclusion of benzyl, acetyl, isopropyl, and trifluoromethyl substituents at positions 3, 5, and 7 of galangin to synthesize 12 derivatives (Figure 5). It turned out that when the 3-position hydroxyl group of galangal was benzylated, the activity also fell significantly when the 3rd and 7th hydroxyl groups of the parent nucleus underwent benzyl substitution, showing that the retention of the 7-position hydroxyl group was crucial for activity. However, more research on the structure–activity correlations is required because there are not many structural modifiers. 

**Scheme 6:**
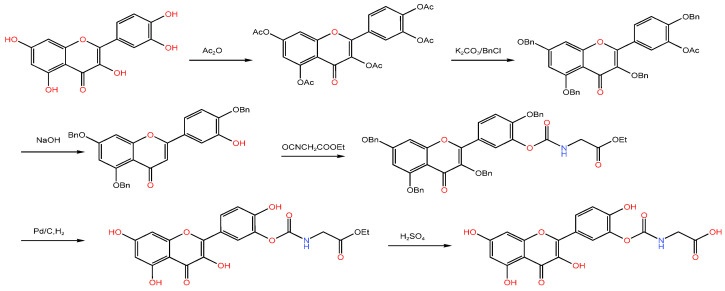
QC12 synthesis path.

**Scheme 7:**
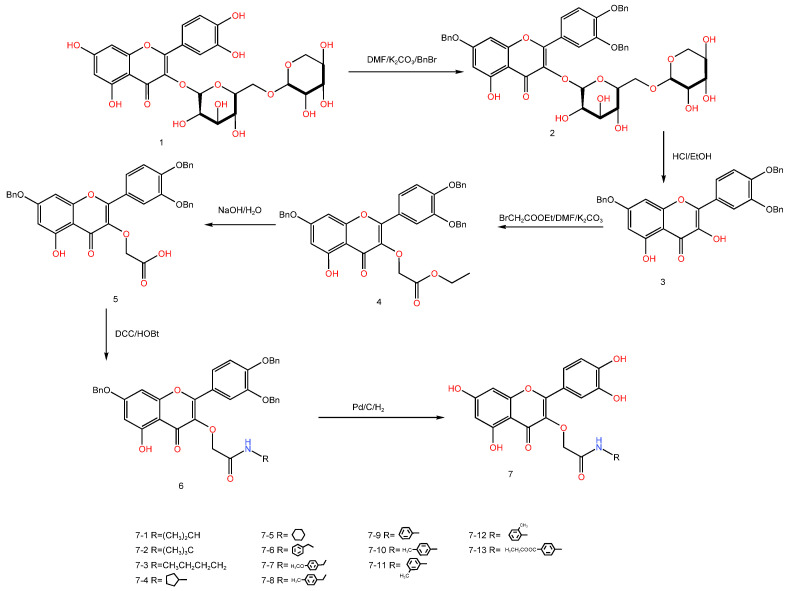
Route of synthesis of quercetinamide derivatives.

Additionally, studies have shown that quercetin in licorice has chemopreventive and antitumor effects, and it has inhibitory effects on human prostate cancer [31], breast cancer [32], lung cancer [33], lymphoma [34], ovarian cancer [35], and other malignant tumor cells; however, because quercetin is a planar molecule, its spatial structure is relatively tightly stacked, and it is not easily dispersed by solvents or solutes, making its water solubility poor (<7 μg/mL); this affects its pharmacokinetic properties, and the bioavailability after entering the human body is low (the peak plasma concentration is only 0.13~7.60 μmol/L) [36], thereby restricting the clinical use of quercetin as an anticancer medication, making its structural modification of particular importance. To summarize the study development of structural modification of quercetin in antitumor in recent years, the following three aspects are mainly considered:

(1) Hydroxyl esterification: Because quercetin has a total of five hydroxyl groups, its hydroxyl groups are typically selectively esterified. Mulholland, P. J et al. [37] and Golding, B. T et al. [38] used quercetin as a launching material, using it in different positions of hydroxyl groups to react differently, selectively maintaining hydroxyl groups to obtain compound **4**, and then adjusting it with ethyl isocyanatoacetate to obtain compound **5**. The synthesis of QC12 is shown in Scheme 6. After compound **5**, dehydroxyprotector undergoes ester hydrolysis under acidic conditions to produce 3′-(N-carboxymethyl) carbamoyl-3, 4′, 5, 7-tetrahydroxyflavone (QC12), the derivative introduces glycine groups to increase its water solubility. Informal phase I clinical trials demonstrate that QC12 can inhibit the growth of human ovarian cancer A2780 cells and block cell proliferation in the late S stage and early G2, making it a new water-soluble anti-cancer lead compound (Scheme 6).

(2) Hydroxy ether formation: Liu, Q. et al. [39] used rutin as the starting material, followed by the Williamson ether reaction, amidation reaction, and Pd/C catalytic hydrodebenzyl to produce 13 quercetinamide derivatives (Scheme 7). When this series of derivatives was tested for antitumor activity, the results revealed that the antitumor activity had increased, with compound **7**–**13** having the greatest inhibitory effect on human esophageal squamous cell carcinoma cells EC109 (IC50 = 10.25 μmol L^−1^) was significantly superior to the parent drug quercetin (IC50 = 31.884 μmol·L^−1^) and 5-FU (IC50 = 41. 738 μmol·L^−1^). 

(3) Formation of metal complexes: Quercetin is a particularly powerful metal chelating agent that can coordinate with a variety of metal ions to form complexes owing to its distinctive planar chemical structure, the presence of hydroxyl groups, and the presence of carbonyl groups. In relation to research by Ksenija et al. [40], quercetin lanthanum complexes have a potent cytotoxic effect on human cervical cancer cell lines (Hela) at concentrations between 100 and 1000 mmol mL^−1^ when exposed for a duration of three hours. Low-dose complexes will have the same biological effect on tumor cells without the negative side effects associated with high drug concentrations, making them potential antitumor medications.

#### 2.1.3. Isoflavones

**Figure 6:**
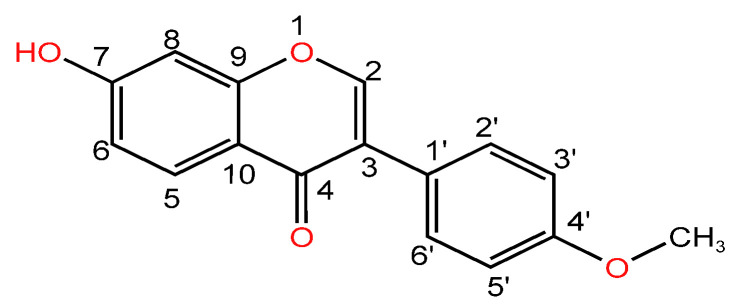
The structure of formononetin.

**Figure 7:**
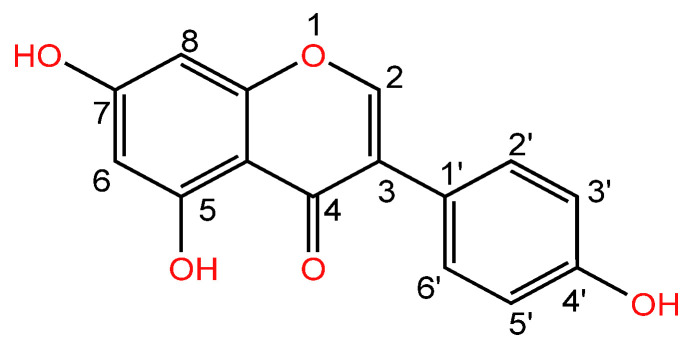
The structure of genistein.

Isoflavones are important components in licorice, with phytoestrogen activity, which can inhibit the proliferation of a variety of cancer cells. Now, more licorice isoflavones have been discovered, primarily the following 37 compounds (Table 3), and the structural alteration of their antitumor components, formononetin (Figure 6) and genistein (Figure 7), is being researched more.

**Scheme 8:**
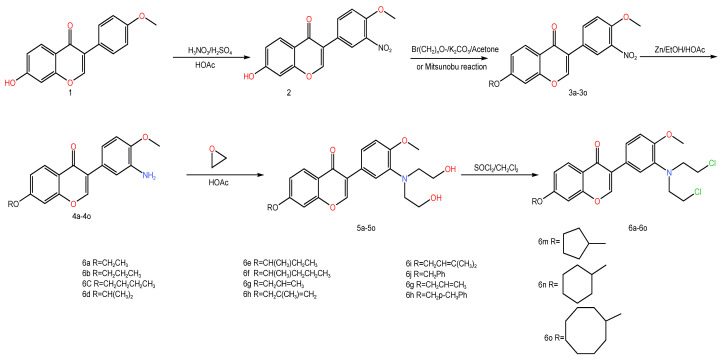
Synthetic route of nitrogen mustard derivatives.

**Scheme 9:**
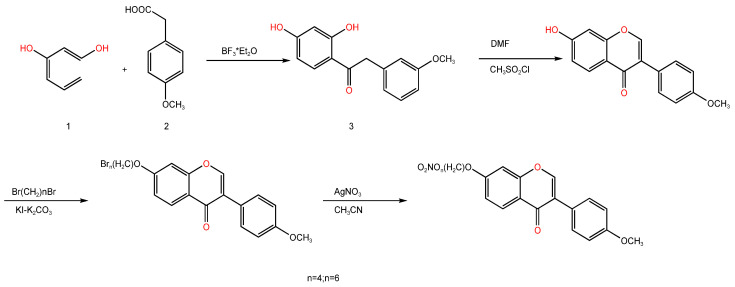
Synthetic route of NO donor derivatives of formononetin.

As an important class of antitumor drugs, nitrogen mustard derivatives are the general term of β-chloroethylamine compounds, including mono β-chloroethanamine and bis β chloroethanamine [61]. At present, the synthesis is relatively simple, the cost is low, and there are many nitrogen mustard drugs that have been used in clinical practice, but there are still shortcomings such as large adverse reactions, low treatment efficiency, poor selectivity, etc. Hu, K et al. [62] produced 15 nitrogen mustard derivatives of frmononetin with frmononetin as the mother in a straightforward five-step reaction involving nitrification, phenolic hydroxyetherification, reduction, amino substitution, and chlorine substitution. He then tested the target compounds’ effects on the human cancer cell lines HCT-116, DU-145, Hela, and SGC-7901 for their ability to inhibit tumor growth, and in vitro antitumor activity experiments showed that compound **6d** had a significant effect on HCT-116 cells, and its IC50 was 3.80 μmol/L, which was stronger than that of the parent drug manganthocyanin (IC50 = 60.97 μmol/L) and the positive control mephalan (IC50 = 20.90 μmol/L), respectively. And, the results can be inferred that the introduction of formononetin in the 7-position hydroxyl group and nitrogen mustard in the 3′ position can enhance the inhibitory effect on tumor cells (Scheme 8). In order to create two novel NO donor-type anthocyanin derivatives, Xin, F et al. [63] connected NO donor fragments on 7-position phenolic hydroxyl groups using the design principle of synergistic prodrugs (Scheme 9).

Likewise, genistein has been highly regarded by the medical community in many different nations as a preventive agent for tumor cells because it has the advantage of not destroying normal cells. Although genistein has a significant first-pass effect and weak lipophilicity and hydrophilicity, this makes it difficult to reach the goal of therapeutic therapy of disorders due to limited bioavailability. As a result, its structure has undergone various changes to increase bioavailability. The following three aspects are where the structural change is currently concentrated.

**Figure 8:**
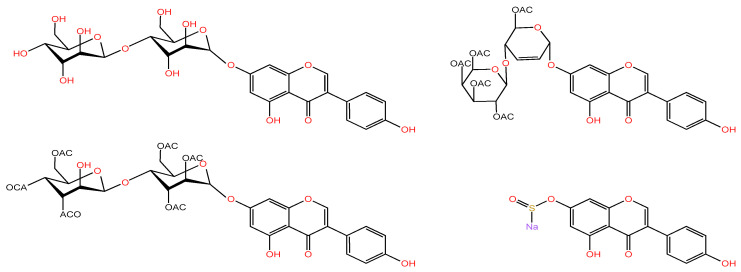
Genistein glycoside derivative structure.

**Figure 9:**
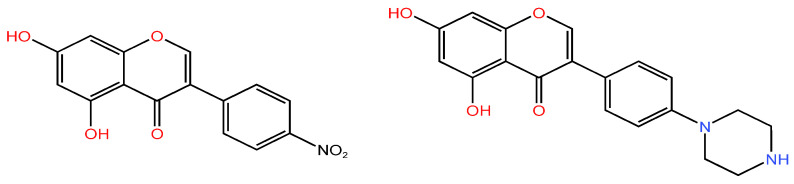
Structural formula of genistein nitro derivative.

(1) Modification of 7-position hydroxyl groups: Based on the lipophilicity and biodegradability of the derivatives, Polkowski, K et al. [64] synthesized an assortment of genistein glycoside derivatives (Figure 8). Studies on the survival of cells in cultivation and cytotoxicity have revealed that hydrophilic glycosides have substantially lower anticancer activity than free glycoside ligands. However, compared to genistein, lipophilic glycosides exhibit a markedly stronger anticancer activity. On the tumor cells Balb/c3T3, Popiołkiewicz’s [65] lipophilic genistein derivatives had a minimum inhibitory concentration of 5 mol/mL, which was a factor of ten lower than genistein’s.

(2) Structural modification of 4′-position hydroxyl group: By adding nitro to the 4′-position of the genistein backbone, Jin, Y et al. [66] constructed genistein nitro derivatives (Figure 9). Results highlighted that, with the exception of the parent compound nitrogenitrin, which exhibited strong antitumor activity, the antitumor effects of the other compounds were less potent than those of the mother compound. It was also evident that the isoflavones’ 7-position hydroxyl group may be the active essential component. 

(3) Structural modification of 2 or 3 hydroxyl groups: Zhang, L et al. [67,68] synthesized more than 50 derivatives of genistein, and they tested the antitumor activity in vitro, in which the 7- and 4′ hydroxyl groups were replaced with ethoxycarbonylformyl groups in all derivatives; the results showed that the antitumor activity had the lowest IC50 (8.5 μmol/L), which was higher than that of the positive control 5-fluorouracil (IC50 = 13.4 μmol/L), which indicated that the replacement hydroxyl group introduced a lipophilic group. It can significantly improve the antitumor activity and lipophilicity of genistein derivatives. 

#### 2.1.4. Chalcones

**Figure 10:**
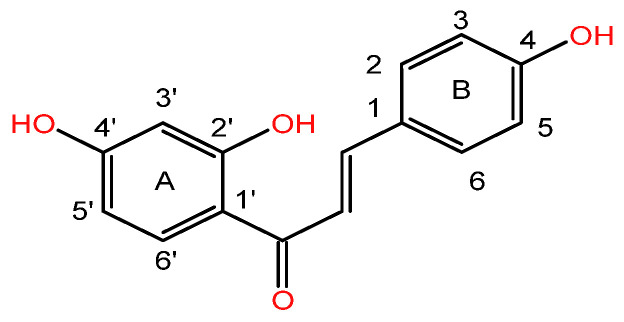
The structure of isoliquiritigenin.

At the moment, there are 24 different types of chalcones (Table 4) found in licorice. While researching the structural modifications of these compounds that make them antitumor active, many researchers discovered that isoliquiritigenin (Figure 10) is connected by two benzene rings, A and B, through the cross-conjugation of propenone. The connected bridge has three atoms, thus facilitating a large degree of selection freedom and a large spatial range of induction fit with the receptor. 

**Scheme 10:**
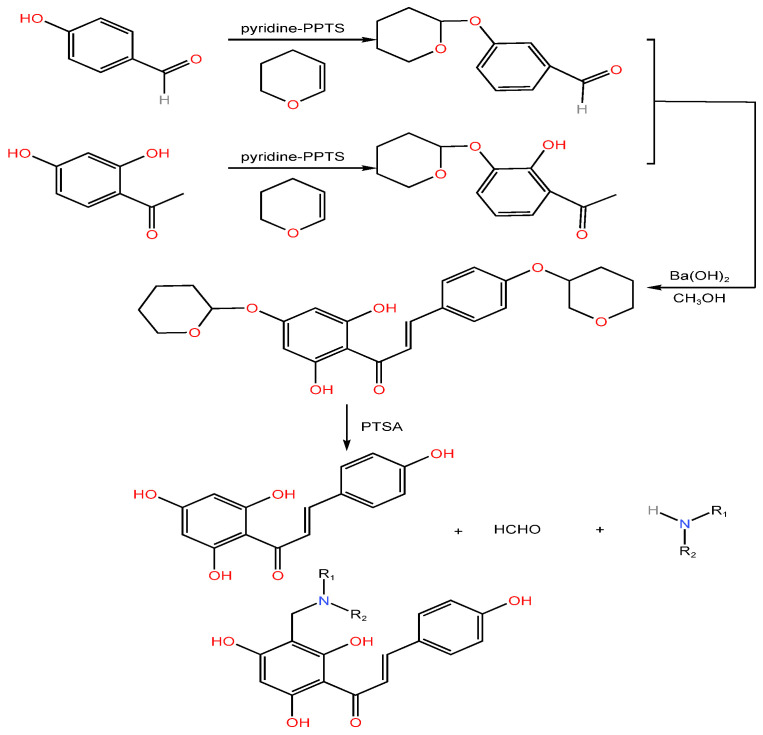
Route of synthesis of isoliquiritigenin Mannich base derivative.

**Scheme 11:**
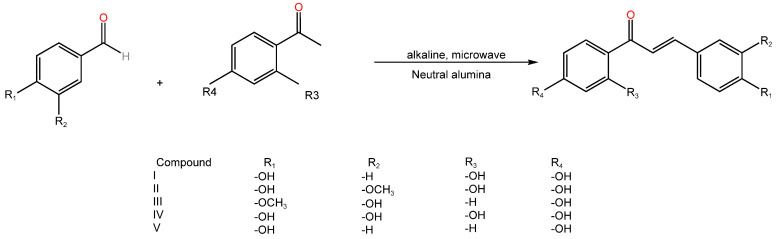
Isoliquiritigenin analogue synthesis route.

Methoxy, -methyl, aryl, and 2′-oxygen-containing groups can be appended to its A ring for boosting its anti-mitotic activity [73,74]. Also, Ma, Y [75] added 10 Mannich groups (Scheme 10) to the A ring 3′-methyl position of 3′-methy lisoliquiritigenin, including dimethylamine, morpholine, cyclohexane, and diisopropylamine. Experiments in mice confirmed that the inhibition rate exceeded than that of isoglycyrrhizin, with diisopropylamine derivatives having the highest inhibition rate. The inhibition rate of isoliquiritigenin S180 tumor cells at 180mg·kg^−1^ was 71.68%. Yang, X et al. [76] and Xu, F et al. [77] discovered that isoliquiritigenin analogues prevented the spread of human cervical cancer cells and that they all had a clear pro-apoptosis effect on SiHa and HeLa cells at a dosage of 100 μg/mL. Activity was increased by adding a single methoxy or hydroxyl group to the isoliquiritigenin B ring’s third position. Although still powerful, the activity of the reduction of one hydroxyl group in the second position of the A ring is less than that of isoliquiritigenin. One hydroxyl group’s activity was decreased at the second position of the A ring, one hydroxyl group was added to the third position of the B ring, and one methoxy group’s activity was added to the fourth position, showing that the hydroxyl groups had been added. It was demonstrated that 3-methoxy isoliquiritigenin had a better apoptotic impact than isoliquiritigenin in the experiments on averting the proliferation of human liver cancer Bel-7402, and the highest possible apoptosis rate could reach 93.5% and 85%, respectively (Scheme 11).

#### 2.1.5. Dihydroflavonoids

There has been little research conducted on the structural modification of antitumor activities for the following 22 dihydroflavonoids (Table 5) that typically occur in licorice. 

**Scheme 12:**
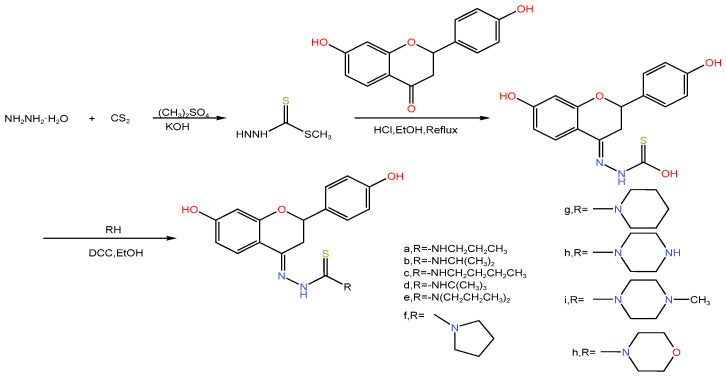
Synthetic route of glycyrrhizin thiourea derivatives.

Liquiritigenin is a flavonoid found in licorice roots. Ren, J et al. [83] discovered that glycyrrhizin along with methyl hydrazinodithioformate were refluxed in ethanol under acidic conditions to form hydrazone, and the methyl dithioformate group was hydrolyzed to thioformate, which was subsequently combined with the corresponding amines under the catalysis of dicyclohexylcarbodiimide (DCC) to obtain a series of glycyrrhizin thiourea derivatives (Scheme 12); the authors also evaluated its effect on human leukemia cells (K562), cytotoxic activity of seven tumor cell lines of human prostate cancer cells (DU-145), human gastric cancer cells (SGC-7901), human colon cancer cells (HCT-116), human breast cancer cells (MCF-7), human liver cancer cells (HepG2), human cervical cancer cells (Hela), and a normal cell line of human renal epithelial cells (293); and, in vitro anticancer activity indicates that most derivatives have certain activity against K562 and DU-145, and from the structure–activity relationship, the authors theorize that the introduction of an amithiourea group at the 4-C position of glycyrrhizin can improve anticancer activity.

### 2.2. Triterpenoids

**Figure 11:**
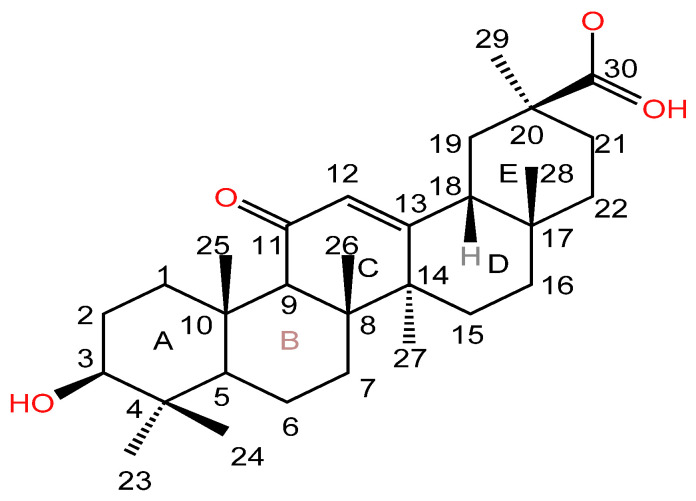
The structure of glycyrrhetinic acid.

Nowadays, licorice mostly contains the following 29 triterpenoid components (Table 6), and glycyrrhetinic acid (Figure 11) is being explored more in relation to the structural modifications of the following chemicals that have antitumor activity.

Glycyrrhetinic acid is usually structurally modified to change the activity due to the weak antitumor activity of the parent nuclear structure. According to a survey of the pertinent scientific literature, the structural change in glycyrrhetinic acid that gave it its antitumor effect focuses mostly on four different regions: the A ring modification, the C ring modification, the E ring modification, and the multi-ring modification. The hydroxyl group at position C-3 on the A ring, the unsaturated carbonyl group at position C-11 on the C ring, and the carboxyl group at position C-30 on the E ring all contain functional groups that are most appropriate for modification.

**Scheme 13:**
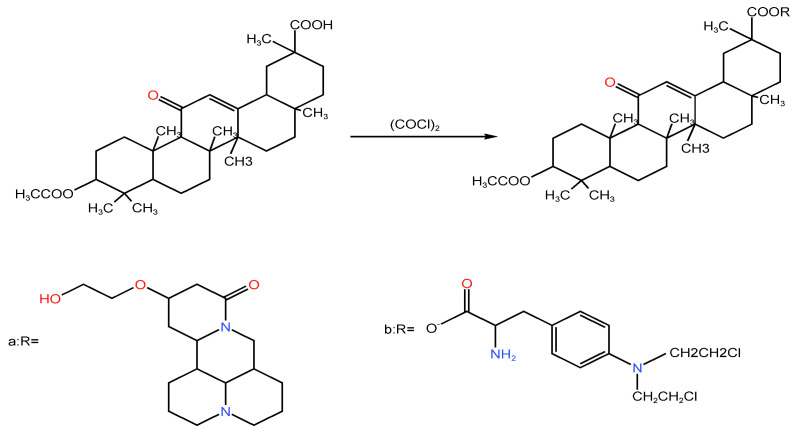
Synthetic route of acetylated derivatives of glycyrrhetinic acid.

**Scheme 14:**
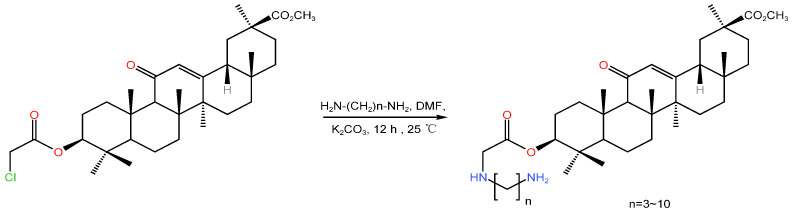
Route of synthesis of aminoalkyl derivatives of glycyrrhetinic acid.

**Figure 12:**
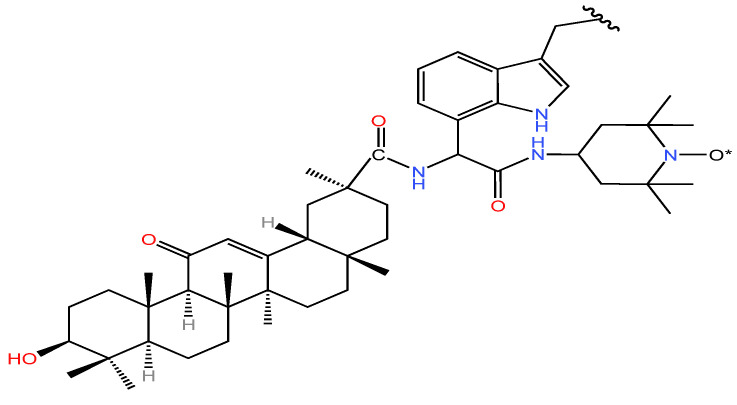
The structure of compound **6j**.

(1) A ring structure modification: In accordance with the drug stitching principle, the hydroxyacetylation at glycyrrhetinic acid C-3 was linked to matrine and melphalan under the action of (COCl)_2_, respectively, and two new glycyrrhetinic acid acetylated derivatives, a and b, were synthesized (Scheme 13); and, the antitumor activity in vivo showed that compounds a and b had a significant inhibitory effect on hepatoma cell SMMC-7721, with IC50 values of 85.7 and 178.2 μmol/L, respectively, and animal experiments showed that they were almost non-toxic to normal liver cells. It is expected to be a candidate for liver-targeted therapy [88]. In addition, in order to improve the polarity of glycyrrhetinic acid, Csuk, R et al. [89] prepared different glycyrrhetinic acid C-3 aminoalkyl derivatives (Scheme 14), and these derivatives were tested for antitumor activity; the results showed that compared with glycyrrhetinic acid, all amino compounds showed significant activity, and the activity changed with the change in carbon number, and when the number of carbon atoms was 6, the activity was the highest, and the IC50 was 0.6–3.0 uM. 

(2) C ring modification: The structural modification research of the C ring mainly focuses on the carbonyl group at the C-11 position; Fiore, C et al. [90] and Mauro, S et al. [91] believe that the carbonyl group at the C-11 position is the main cause of the apoptotic activity of GA derivatives, but the study of Csuk, R et al. [89] shows that the existence of C-11 carbonyl group is not directly related to the apoptotic activity of the compound; therefore, the relationship between the existence of C-11 carbonyl group and apoptotic activity needs to be further studied.

(3) E ring modification: Liu, Y et al. [92] synthesized a series of glycyrrhetinic acid derivatives by introducing nitro radicals and amino acid fragments into glycyrrhetinic acid, and compound **6j** (Figure 12) with tryptophan amino group and piperidine nitro group in these derivatives has the strongest cytotoxicity (GI50 is 13.7~15.0 μM), five times stronger than glycyrrhetinic acid, and it is speculated that adding nitro and amino acid fragments to the C-30 carboxyl group of glycyrrhetinic acid may help improve its cytotoxicity. 

(4) Polycyclic modification: The polycyclic modification of glycyrrhetinic acid is mainly concentrated on the A, C and E rings, among which the structural modification of A ring and E ring is the most prominent. Shen, L.H et al. [93] found that modifying both the C-3 hydroxyl group and C-30 carboxyl group had antitumor activity, and they found that when the carbon chain length of the linker group was 2~4, the activity increased with the extension of the carbon chain length, and when the carbon chain length of the linker group was 5, the activity decreased. Additionally, they discovered that the compound’s anticancer effect was increased when C-3 and C-30 were present with nitrate moieties simultaneously.

## 3. Conclusions and Outlook

In recent times, some advancements have been made in the study and development of licorice-based antitumor pharmaceuticals, but there are still several issues that limit the creation and application of the therapeutic substance. The three main challenges are the ones that follow: (1) Liquiritin and glycyrrhizic acid ammonium salt have been designated as standard compounds to determine the quality of licorice in the Chinese Pharmacopoeia 2020 edition, but given that many other compounds have been found to have good pharmacological activity, it is advised to update the quality evaluation method to create more thorough, systematic, and standardized quality evaluation standards for licorice herbs, with the objective being to increase the efficiency of their utilization. (2) The research on the antitumor mechanisms is discussed from multiple viewpoints, the research is fragmented, and there is a lack of systematic and comprehensive analysis. The chemical structure modification of licorice antitumor drugs focuses mainly on flavonoids and triterpenoids, while carbohydrates and coumarins are less studied. (3) Licorice can detoxify hundreds of poisons and has the ability to harmoniously combine and detoxify different medications. Licorice is advantageous to the drug resistance brought on by anticancer medications and the sensitivity of tumor cells, according to fundamental research findings, although there are few clinical studies and insufficient links to basic research. As a result, future research on and the use of licorice will make use of pertinent network pharmacology, bioinformatics, and other converged technologies.

## Figures and Tables

**Table 1:** Licorice flavonoid compounds.

Serial Number	Name of the Compound	Molecular Formula	Reference
1	schaftoside	C_26_H_28_O_14_	[9]
2	vicenin-2	C_27_H_30_O_15_	[9]
3	isoshaftoside	C_26_H_28_O_14_	[9]
4	shaftoside	C_26_H_28_O_14_	[9]
5	isoviolanthin	C_27_H_30_O_14_	[9]
6	isoschaftoside	C_26_H_28_O_14_	[10]
7	violanthin	C_27_H_30_O_14_	[10]
8	apigenin	C_15_H_10_O_5_	[11]
9	licoflavon	C_20_H_18_O_4_	[12]
10	kumatakenin	C_17_H_14_O_6_	[13]
11	naringin	C_27_H_32_O_14_	[14]
12	isoangustone A	C_25_H_26_O_6_	[15]
13	genkwanin	C_16_H_12_O_5_	[16]
14	uralenol	C_21_H_18_O_7_	[15]

**Table 2:** Licorice flavonol compounds.

Serial Number	Name of the Compound	Molecular Formula	Reference
1	licoflavonol	C_20_H_18_O_6_	[24]
2	galangin	C_15_H_10_O_5_	[25]
3	kaempferol	C_15_H_10_O_6_	[16]
4	isoquercetin	C_21_H_20_O_12_	[16]
5	astragalin	C_21_H_20_O_11_	[16]
6	narcissin	C_28_H_32_O_16_	[26]
7	isoquercitrin	C_21_H_20_O_12_	[27]
8	quercetin	C_15_H_10_O_7_	[28]

**Table 3:** Licorice isoflavone compounds.

Serial Number	Name of the Compound	Molecular Formula	Reference
1	licoisoflavone A	C_20_H_18_O_6_	[41]
2	eurycarpin A	C_20_H_18_O_5_	[42]
3	formononetin	C_16_H_12_O_4_	[43]
4	eurycarpin B	C_20_H_16_O_5_	[42]
5	gancaonin G	C_21_H_20_O_5_	[36]
6	licoricone	C_22_H_22_O_6_	[44]
7	afromosin	C_17_H_14_O_5_	[45]
8	odoratin	C_17_H_14_O_6_	[46]
9	prunetin	C_16_H_12_O_5_	[47]
10	genistein	C_15_H_10_O_5_	[48]
11	ononin	C_22_H_22_O_9_	[42]
12	licoagroside A	C_23_H_25_O_12_	[49]
13	lupiwighteone	C_20_H_18_O_5_	[50]
14	2,3-dehydrokievitone	C_20_H_18_O_6_	[51]
15	gancaonin H	C_25_H_24_O_6_	[52]
16	glycyroside	C_27_H_30_O_13_	[53]
17	calycosin	C_16_H_12_O_5_	[54]
18	glabrene	C_20_H_18_O_4_	[55]
19	2′-hydroxyisolupalbigenin	C_25_H_26_O_6_	[56]
20	licoricone	C_22_H_22_O_6_	[55]
21	glicoricone	C_21_H_20_O_6_	[55]
22	3′,6′-diethoxyfluorescein	C_24_H_20_O_5_	[55]
23	3,7-dimethyl licoflavonol	C_22_H_24_O_6_	[15]
24	glabrizoflavone	C_20_H_18_O_6_	[57]
25	6,8-di-(dimethylallyl)-genistein	C_25_H_26_O_5_	[58]
26	glyzarin	C_18_H_14_O_4_	[59]
27	isoglabrone	C_20_H_16_O_5_	[60]
28	parvisoflavone B	C_20_H_16_O_6_	[56]
29	3′,4′-dihydroxy-7-methoxyisoflavone	C_16_H_12_O_5_	[10]
30	derrone	C_20_H_16_O_5_	[51]
31	parvisoflavones A	C_20_H_16_O_6_	[51]
32	isoglabrone	C_20_H_16_O_5_	[51]
33	glycybridin D	C_25_H_26_O_4_	[55]
34	glycybridin E	C_25_H_24_O_4_	[55]
35	glycybridin J	C_21_H_22_O_6_	[55]
36	semilicoisoflavone B	C_20_H_16_O_6_	[42]

**Table 4:** Licorice chalcone compounds.

Serial Number	Name of the Compound	Molecular Formula	Reference
1	isoliquiritigenin	C_15_H_12_O_4_	[55]
2	isoliquiritin	C_21_H_22_O_9_	[12]
3	licuraside	C_26_H_30_O_13_	[12]
4	licochalcone A	C_21_H_22_O_4_	[12]
5	licochalcone B	C_16_H_14_O_5_	[12]
6	neoisoliquiritin	C_21_H_22_O_9_	[12]
7	echinatin	C_16_H_14_O_4_	[69]
8	glycybridin A	C_20_H_20_O_5_	[51]
9	glycybridin B	C_20_H_22_O_5_	[51]
10	glycybridin C	C_25_H_30_O_5_	[51]
11	kanzonol Y	C_25_H_30_O_5_	[51]
12	kanzonol C	C_25_H_28_O_4_	[51]
13	kanzonol B	C_20_H_18_O_4_	[51]
14	licoagrochalcone A	C_20_H_20_O_4_	[51]
15	paratocarpins B	C_25_H_26_O_4_	[51]
16	isobavachalcone	C_20_H_20_O_4_	[51]
17	chalconaringenin4-O-glucoside	C_21_H_22_O_10_	[70]
18	3-hydroxylicochalcone E	C_21_H_22_O_5_	[70]
19	licoagrochalcone D	C_21_H_22_O_5_	[70]
20	[6″,6″-dimethylpyran-(2″,3″4,5)]-3′-γ,γ-dimethylally-2′,3,4′-thrihydroxychalcone	C_25_H_26_O_5_	[71]
21	licoagrochalcone C	C_21_H_22_O_5_	[60]
22	kanzonol C	C_25_H_28_O_4_	[60]
23	4-hydroxylonchocarpin	C_20_H_18_O_4_	[72]
24	licoagrochalcone B	C_21_H_20_O_4_	[49]

**Table 5:** Licorice dihydroflavonid compounds.

Serial Number	Name of the Compound	Molecular Formula	Reference
1	glabrol	C_25_H_28_O_4_	[78]
2	glabranin	C_20_H_20_O_4_	[55]
3	liquiritigenin	C_15_H_12_O_4_	[55]
4	glabranin isomer	C_20_H_20_O_4_	[79]
5	liquiritin	C_21_H_22_O_9_	[12]
6	liquiritin apioside	C_26_H_30_O_13_	[12]
7	pinocembrin	C_15_H_12_O_4_	[80]
8	licoflavanone	C_20_H_18_O_5_	[80]
9	xambioona	C_25_H_24_O_4_	[51]
10	shinflavanone	C_25_H_26_O_4_	[51]
11	(2S)-abyssinone I	C_20_H_18_O_4_	[51]
12	euchrenone a5	C_25_H_26_O_4_	[51]
13	naringenin	C_15_H_12_O_5_	[51]
14	abyssinone II	C_20_H_20_O_4_	[51]
15	naringin	C_27_H_32_O_14_	[51]
16	pinocembroside	C_21_H_22_O_9_	[9]
17	shinflavone	C_25_H_26_O_4_	[55]
18	licoagrodin	C_45_H_44_O_9_	[81]
19	neoliquiritin	C_21_H_22_O_9_	[81]
20	3′-prenylnaringenin	C_21_H_22_O_9_	[82]
21	cyclolicoflavanone	C_20_H_20_O_5_	[82]
22	glabranine	C_20_H_20_O_4_	[82]

**Table 6:** Licorice triterpene compounds.

Serial Number	Name of the Compound	Molecular Formula	Reference
1	glycyrrhizic acid	C_42_H_62_O_16_	[84]
2	glycyrrhetinic acid	C_30_H_46_O_4_	[84]
3	glycyrrhizin	C_42_H_62_O_16_	[84]
4	glycyrrhetol	C_30_H_48_O_3_	[12]
5	glabric acid	C_30_H_46_O_5_	[12]
6	liquoric acid	C_30_H_44_O_5_	[12]
7	glabrolide	C_30_H_44_O_4_	[12]
8	isoglabrolide	C_30_H_44_O_4_	[12]
9	ursolic acid	C_30_H_48_O_3_	[80]
10	betulin	C_30_H_50_O_2_	[80]
11	lupeol	C_30_H_50_O	[80]
12	licorice-saponin M3	C_48_H_74_O_19_	[85]
13	licorice-saponin N4	C_54_H_84_O_23_	[85]
14	licorice-saponin O4	C_54_H_84_O_24_	[85]
15	licoricesaponin G2	C_42_H_62_O_17_	[85]
16	18α-licorice-saponin G2	C_42_H_62_O_17_	[85]
17	macedonoside A	C_42_H_62_O_17_	[85]
18	29-hydroxy-glycyrrhizin	C_42_H_62_O_16_	[85]
19	licorice-saponin A3	C_48_H_72_O_21_	[85]
20	24-hydroxy-licorice-saponin A3	C_48_H_72_O_22_	[85]
21	22β-acetoxyl-glycyrrhizin	C_44_H_64_O_18_	[85]
22	licorice-saponin H2	C_42_H_62_O_16_	[12]
23	licorice-saponin R3	C_48_H_74_O_20_	[86]
24	licoricesaponin S3	C_48_H_75_O_20_	[86]
25	uralsaponin C	C_42_H_64_O_16_	[86]
26	18α-glycyrrhizic acid	C_42_H_62_O_16_	[10]
27	18β-glycyrrhizic acid	C_42_H_62_O_16_	[10]
28	licorice-saponin P2	C_42_H_62_O_17_	[10]
29	betulic acid	C_30_H_48_O_3_	[87]

## Data Availability

Not applicable.
